# Antitumor action of the peroxisome proliferator-activated receptor-γ agonist rosiglitazone in hepatocellular carcinoma

**DOI:** 10.3892/ol.2015.3554

**Published:** 2015-07-30

**Authors:** QI-FU BO, XIU-MEI SUN, JIN LIU, XIAO-MEI SUI, GUI-XIN LI

**Affiliations:** Department of Oncology, Affiliated Hospital of Weifang Medical University, Weifang, Shandong 261000, P.R. China

**Keywords:** rosiglitazone, peroxisome proliferator-activated receptor γ, phosphoinositide 3-kinase/protein kinase B, apoptosis, proliferation

## Abstract

The inhibition of apoptosis in cancer cells is the major pathological feature of hepatic carcinoma. Rosiglitazone (RGZ), a ligand for peroxisome proliferator-activated receptor γ (PPAR-γ), has been shown to induce apoptosis in hepatic carcinoma cells. However, the mechanism underlying this effect remains to be elucidated. The present study aimed to investigate the effect of RGZ on cell viability and apoptosis, and its mechanisms in cultured HepG2 cells using MTT assay, flow cytometry and western blotting. The results revealed that treatment with RGZ may attenuate HepG2 cell viability and induce the apoptosis of the cells. The mechanism of RGZ-induced apoptosis involves an increase in the level of activated PPAR-γ (p-PPAR-γ) and a decrease in p85 and Akt expression. In addition, the PPAR-γ antagonist GW9662 suppressed the effect of RGZ in the HepG2 cells. Taken together, the results suggest that RGZ induces the apoptosis of HepG2 cells through the activation of PPAR-γ, suppressing the activation of the PI3K/Akt signaling pathway. Such mechanisms may contribute to the favorable effects of treatment using RGZ in HepG2 cells.

## Introduction

Hepatocellular carcinoma (HCC) is one of the most frequent tumors of the liver, and it is reported to account for 5% of all malignant neoplasms (1,2). The aggressiveness and wide dissemination of HCC frequently leads to mortality in the affected population (3), and despite the development of modern treatment protocols, the incidence and mortality rates of the disease remain high (3). Therefore, investigation into novel methods to improve the treatment and survival of liver cancer patients is essential. The pathogenesis of liver cancer is complex, and studies have demonstrated that uncontrolled proliferation, various ion disorders and resistance to apoptosis are key features of this process (4). However, the exact mechanisms of pathogenesis are unclear, particularly that of apoptosis resistance.

It has been demonstrated that apoptosis, a type of programmed cell death, is vital in the prevention of HCC development (5). The activation of apoptotic pathways is a key mechanism by which HCC cells may be killed, and defects in apoptotic signaling can lead to the drug resistance of these cells (6). Therefore, the induction of apoptosis is considered to be an important method in the assessment of the clinical effectiveness of anti-HCC drugs.

Notably, the phosphoinositide 3-kinase/protein kinase B (PI3K/Akt) pathway, which has been demonstrated to play a critical role in regulating cell growth and cell survival in different systems, has been identified to be involved in the pathogenesis of HCC by inducing the apoptosis resistance of HCC cells (7). The PI3K kinase is composed of a catalytic subunit, p110, and a regulatory subunit, p85; the activation of PI3K depends upon the activation of the p85 subunit. When p85 is activated, it directs signals to phosphorylate Akt, which subsequently provides signals that regulate apoptosis resistance (8). Inhibiting PI3K/Akt signaling has been reported to induce apoptosis in HCC cells (9,10).

For the past several decades, rosiglitazone (RGZ), an agonist of peroxisome proliferator-activated receptor γ (PPAR-γ), has been extensively used in clinical practice due to its important regulatory role in energy homeostasis, and lipid and glucose metabolism (11). PPAR-γ is widely distributed in HCC cells (12). It has been demonstrated that RGZ regulates the activity of transcription factors essential for apoptosis, and it has also been used to induce apoptosis in leukemia cells and lung cancer cells though the PI3K-Akt signaling pathway (13,14). However, the effect of RGZ on HCC cells is yet to be elucidated. Therefore, the present study investigated its effects in the classical human HCC HepG2 cell line (15) to address this question.

## Materials and methods

### 

#### Main reagents

RGZ, purchased from Cayman Chemical Company (Ann Arbor, MI, USA), was dissolved in dimethyl sulfoxide (DMSO) and stored at −20°C. GW9662, a PPAR-γ antagonist, was also purchased from Cayman Chemical Company. The Annexin V-fluorescein isothiocyanate (FITC)/propidium iodide (PI) Apoptosis Detection Kit was purchased from R&D Systems, Inc. (Minneapolis, MN, USA).

#### Cell culture

The human HCC HepG2 cell line was obtained from the American Type Culture Collection (Rockville, MD, USA). The cells were plated at a density of 2×10^5^ cells/cm^2^ and cultured in Dulbecco's modified Eagle's medium containing 10% fetal bovine serum (Gibco-BRL Life Technologies, Grand Island, NY, USA) and penicillin/streptomycin (100 U/l; Wuhan Goodbio Technology Co. Ltd., Wuhan, China), at 37°C in a humidified atmosphere of 5% CO_2_ and 95% air.

#### Cell viability rate

The cell viability rate was assessed using the microculture tetrazolium method. The cells were plated at a density of 3×10^4^/cm^2^ in 96-well plates. The HepG2 cells contained 6 parallel wells were divided into the control, RGZ and GW9662 groups. They were incubated with DMSO, RGZ and RGZ+GW9662 for 72 h, respectively, and 10 µl MTT working solution was added, followed by continuous incubation for 4 h. All culture medium supernatant was removed from each well following centrifugation at 3,000 × g, and replaced with 100 µl DMSO. Following thorough solubilization, the absorbance (A) of each well was measured using a microculture plate reader at 570 nm. The cell inhibitory rate was calculated according to the following formula: Inhibitory rate = 100 × (A_control group_ - A_treated group_) / A_control group_).

#### Flow cytometric (FCM) analysis of cell apoptosis

Apoptosis was assessed using an Annexin V-FITC/PI Apoptosis Detection Kit. For FCM analysis, 2×10^5^ cells/well were treated with different concentrations of RGZ (0, 20, 40, 80 µg/ml) or GW9662 (0, 5, 10, 20 µg/ml). The cells were subsequently collected, pelleted and washed with phosphate-buffered saline (PBS) prior to fixing overnight at −20°C in 75% ethanol. The cells were washed again with PBS, resuspended and treated with RNase (200 mg/l) for 30 min at 37°C, prior to incubation with 20 mg/l PI in the dark for 15 min. The suspension was passed through a nylon mesh filter and underwent FCM analysis (FACSort^™^; Becton-Dickinson, Franklin Lakes, NJ, USA). All data were collected, stored and analyzed by LYSIS II software (Becton-Dickinson). The experiments were repeated three times, and the results are presented as the mean ± standard deviation (SD).

#### Western blotting

Total proteins from HepG2 cells in the three groups were separated by 12% SDS-PAGE (Wuhan Goodbio Technology Co. Ltd.), and the separated proteins were electrotransferred onto nitrocellulose membranes (Wuhan Goodbio Technology Co. Ltd.) using a Trans-Blot® Turbo™ Transfer System (Bio-Rad Laboratories, Inc., Hercules, CA, USA). The membranes were first blocked with 5% non-fat milk for 2 h at room temperature, prior to incubation with the following primary monoclonal anti-human antibodies: PPAR-γ, activated PPAR-γ (p-PPAR-γ; rabbit; 1:500; cat. no. ab195925; Abcam, Cambridge, UK), PPAR-γ (1:1,000; rabbit; cat. no. ab59256; Abcam) p85, p-p85, Akt, p-Akt, caspase 3 (rabbit; 1:500; cat. no. ab32351; Abcam), cleavage-caspase 3 (rabbit; 1:500; cat. no. ab2302; Abcam), Bax (rabbit; 1:1,000; cat. no. ab7977; Abcam), Bcl-2 (mouse; 1:500; cat. no. ab117115; Abcam), p-p85 (rabbit; 1:1,000; cat. no. 4228S; Cell Signaling Technology, Inc., Danvers, MA, USA), p85 (rabbit; 1:500; cat. no. 4292S; Cell Signaling Technology, Inc.), p-Akt (rabbit; 1:500; cat. no. 4060S; Cell Signaling Technology, Inc.), Akt (rabbit; 1:1,000; cat. no. 4685S; Cell Signaling Technology, Inc.) and β-actin (rabbit; 1:3,000; cat. no. ab6276; Abcam). Anti-rabbit antibody conjugated to horseradish peroxidase (Jackson ImmunoResearch Laboratories, West Grove, PA, USA) was used as the secondary antibody. β-actin was used as an intrinsic quality control. The bands were incubated in ECL Plus reagent (Amersham, Piscataway, NJ, USA) and chemiluminescence was detected on BioMax MR Film (Kodak, Rochester, NY, USA). The density of the bands was quantified using Labworks image acquisition and analysis software (UVP LLC, Upland, CA, USA) (16).

#### Statistical analysis

All experiments were performed in triplicate, and the results are expressed as the mean ± SD. For the statistical analysis, Student's t-tests were performed using SPSS software, version 12.0 (SPSS, Inc., Chicago, IL, USA). P<0.05 was considered to indicate a statistically significant difference.

## Results

### 

#### RGZ significantly inhibits the cell viability of HepG2 cells

**Figure 1.**
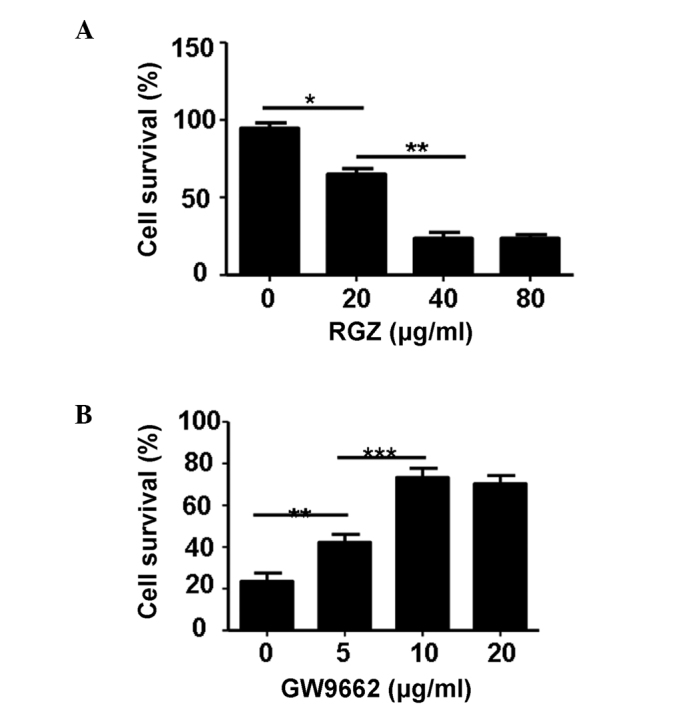
RGZ attenuates the cell viability of HepG2 cells. (A) After treatment with various concentrations of RGZ for 72 h, the cell viability was determined by MTT assay. (B) The cell viability of HepG2 cells treated with various concentrations of GW9662 and RGZ (40 µg/ml) was also determined by MTT assay. Experiments were conducted in triplicate and results are presented as the mean ± standard deviation. *P<0.05, **P<0.01 and ***P<0.001. RGZ, rosiglitazone.

The cytotoxic effect of RGZ on HepG2 cells was determined following incubation with varying concentrations of RGZ by MTT assay. As shown in Fig. 1A, RGZ treatment significantly attenuated the cell viability of HepG2 cells, with a concentration of 40 µg/ml at 72 h producing the optimal effect (P<0.01). Inhibition of cell viability by RGZ was dose-dependent from 0–40 µg/ml. In order to further demonstrate that the cytotoxic effect on HepG2 cell viability was caused by RGZ, the cells were treated with various concentrations (0, 5, 10 and 20 µg/ml) of PPAR-γ antagonist, GW9662, plus RGZ (40 µg/ml). As shown in Fig. 1B, GW9662 significantly attenuated the cytotoxic effect of RGZ in the HepG2 cells. The optimal concentration of GW9662 to attenuate the cytotoxic effect of RGZ was 10 µg/ml (P<0.001).

#### RGZ induces the apoptosis of HepG2 cells

**Figure 2.**
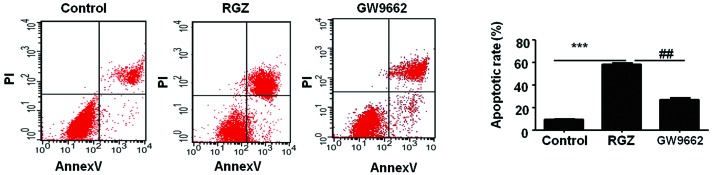
Apoptosis detection by flow cytometric (FCM) analysis. After the cells were treated with RGZ and GW9662, FCM analysis was used to detect the apoptotic rate. The cells were stained with propidium iodide prior to analysis. Experiments were repeated three times, and results are presented as the mean ± standard deviation. ^##^P<0.01; ***P<0.001. RGZ, rosiglitazone; AnnexV, Annexin V.

The most effective concentrations of RGZ and GW9662 were used to further analyze the effect of RGZ on the HepG2 cell lines. The effect of RGZ on the apoptosis of the HepG2 cell lines was examined by FCM analysis. Compared with the HepG2 cells from the control group, the RGZ-treated cells exhibited a higher rate of apoptosis (P<0.001). Notably, the HepG2 cells in the GW9662-treated group exhibited a 1.3-fold lower rate of apoptosis compared with the cells in the RGZ-treated group (P<0.01). These results indicated that the administration of RGZ may significantly induce apoptosis in the HepG2 cells (Fig. 2).

**Figure 3.**
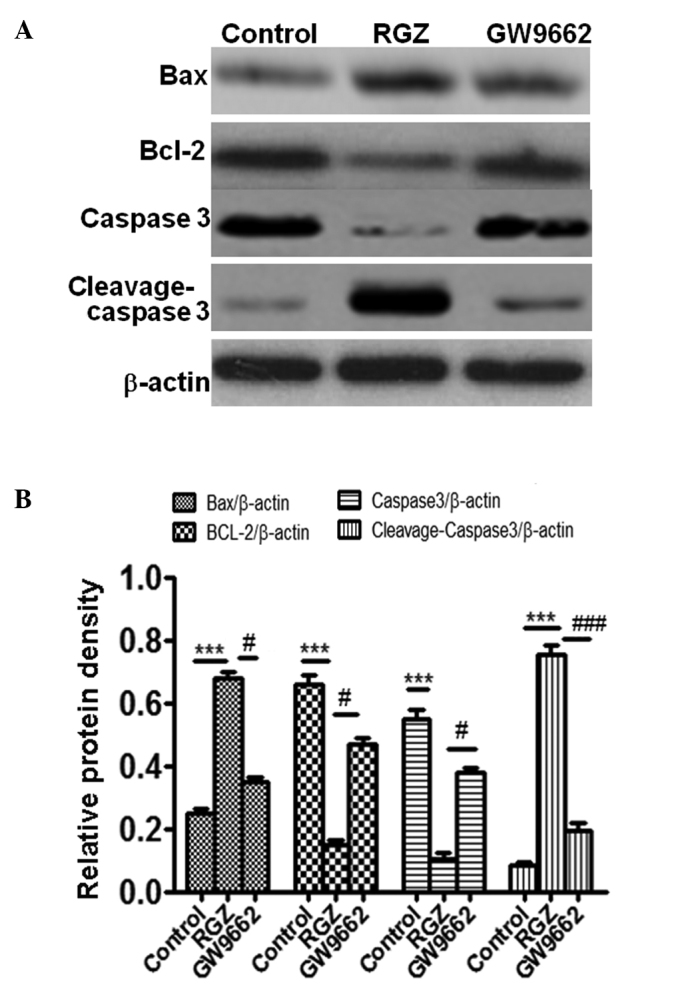
RGZ treatment increases the expression of Bax, cleavage-caspase 3 and decreases the expression of Bcl-2 and caspase 3. Western blot analysis was employed to assess Bax, Bcl-2, caspase 3 and cleavage-caspase 3 expression. (A) Representative western blot results. (B) Semi-quantitative analysis of the cells studied in each group. The relative amount of Bax, Bcl-2, caspase 3 and cleavage-caspase 3 in each group was normalized to β-actin. ^#^P<0.05 and ^###^P<0.001, RGZ vs. GW9662; ***P<0.001, RGZ vs. control. RGZ, rosiglitazone.

It has been previously demonstrated than Bax/Bcl-2 protein are associated with apoptosis (17,18). In order to further demonstrate the effect of RGZ on apoptosis, the expression of Bax and Bcl-2 was examined by western blotting in RGZ-treated HepG2 cells. As shown in Fig. 3, RGZ-treated cells exhibited increased expression of Bax and reduced expression of Bcl-2 compared with the cells in the control (P<0.001) and GW9662-treated (P<0.05) groups. This result was a further indication that RGZ was able to induce apoptosis in the HepG2 cells.

Caspase 3 activation has previously been demonstrated to serve an important role in apoptosis (17,18). The present results demonstrated that administration of RGZ can significantly reduce levels of caspase 3 (P<0.001 and P<0.05 compared with the control and GW9662-treated cells, respectively) and increase cleavage-caspase 3 (P<0.001 compared with the which further indicated that RGZ could induce the apoptosis of HepG2 cells (P<0.05).

#### RGZ induces apoptosis through PPAR-γ activation

**Figure 4.**
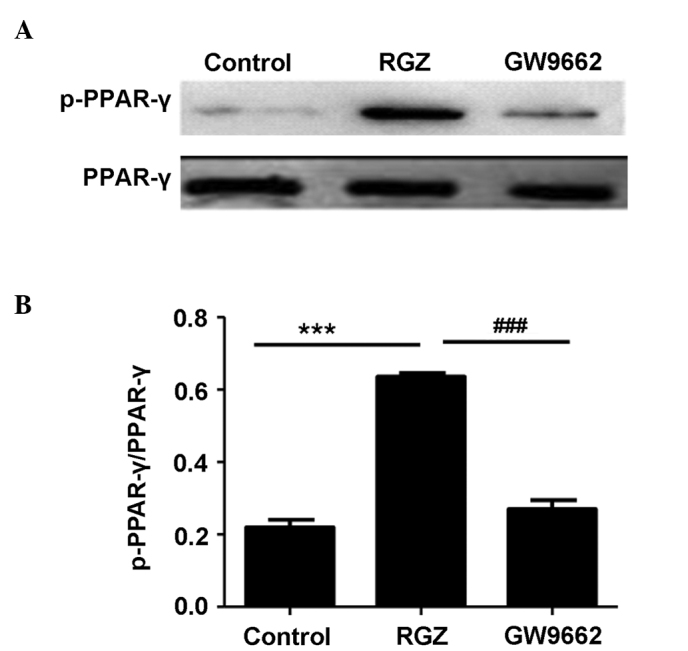
RGZ treatment promotes PPAR-γ activation. Western blot analysis was employed to assess PPAR-γ expression and activation by evaluating the levels of total PPAR-γ and activated PPAR-γ (p-PPAR-γ). (A) A representative result obtained by western blot analysis. (B) Semi-quantitative analysis of cells studied in each group. The relative amount of PPAR-γ and p-PPAR-γ in each group of cells was normalized by β-actin and presented as the ratio of p-PPAR-γ to PPAR-γ. ^###^P<0.01; ***P<0.001. PPAR-γ, peroxisome proliferator-activated receptor-γ; RGZ, rosiglitazone.

As the first step to addressing the underlying mechanisms of the RGZ-induced apoptosis of HepG2 cells, PPAR-γ activation was examined. RGZ is an agonist for PPAR-γ, while GW9662 is an antagonist. Unexpectedly, RGZ and GW9662 administration exerted no perceptible effect on PPAR-γ expression (P>0.05). However, the amount of activated p-PPAR-γ observed in the HepG2 cells following RGZ administration was significantly higher compared with that of the control (P<0.001) and GW9662-treated (P<0.01) cells. These results indicated that RGZ was able to induce PPAR-γ activation, while GW9662 suppressed the effect of RGZ on PPAR-γ activation (Fig. 4).

#### PPAR-γ activation downregulates PI3K/Akt signaling

**Figure 5.**
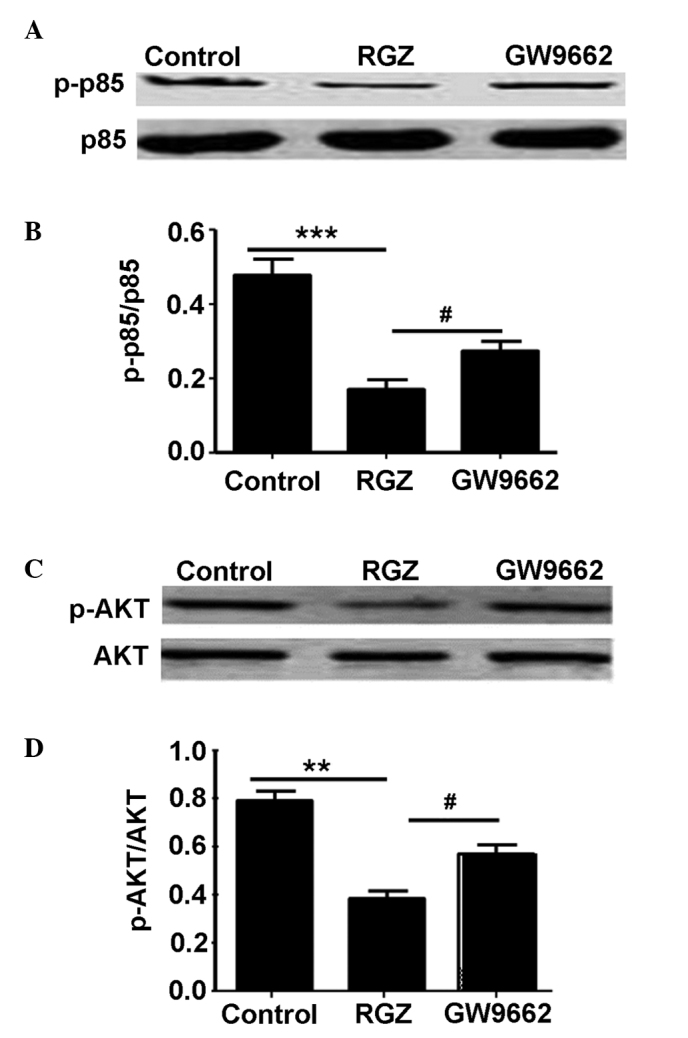
Administration of RGZ suppresses PI3K/Akt signaling activation. The p85 regulatory subunit was used to assess the activity of PI3K, while activated Akt (p-Akt) was employed for evaluating Akt activation. (A) A representative western blot result for p85 and p-p85. (B) Semi-quantitative analysis of cells studied in each group. The relative amount of p85 and p-p85 in each group of cell was normalized by β-actin and presented as the ratio of p-p85 to p85. (C) A representative western blot result for Akt and p-Akt. (D) Semi-quantitative analysis of cells studied in each group. The relative amount of Akt and p-Akt in each group of cell was normalized by β-actin and presented as the ratio of p-Akt to Akt. ^#^P<0.05; **P<0.01; ***P<0.001. RGZ, rosiglitazone; PI3K/Akt, phosphoinositide 3-kinase/protein kinase B.

The aforementioned results prompted the investigation of the impact of PPAR-γ activation on PI3K/Akt signaling, a well-established pathway that has significant implications in HepG2 cells (15). To this end, the activity of the PI3K p85 regulatory subunit was first examined. No significant difference in total p85 levels was detected between the 3 groups of HepG2 cells (P>0.05; Fig. 5A). However, markedly lower levels of p-p85 were noted in the RGZ-treated group compared with the GW9662-treated or control groups (P<0.05 and P<0.001, raspectively; Fig. 5B). As p85 activation provides signals to Akt, Akt activity was subsequently examined. Similar to p85, no difference was observed in total Akt levels between the groups (P>0.05; Fig. 5C), whilst p-Akt levels were significantly lower in the RGZ-treated group compared with the control (P<0.01) or GW9662-treated (P<0.05) groups. Collectively, these data suggested that RGZ treatment increased PPAR-γ activation, and that p-PPAR-γ attenuated PI3K p85 activity, which subsequently decreased Akt activation (Fig. 5D).

## Discussion

Although RGZ has been used extensively in clinical practice in the treatment of diabetes (11), its impact on HCC cells remains to be studied. Therefore, its effects on HepG2 cells were investigated in the present study. RGZ treatment was found to significantly attenuate cell viability and induce apoptosis in HepG2 cells. The effect of RGZ on cell viability was partially dose-dependent, and a concentration of 40 µg/ml was found to produce the greatest effect. GW9662, an antagonist of PPAR-γ, was observed to suppress the effect of RGZ on cell viability in HepG2 cells in a partially dose-dependent manner, with an optimal concentration of 10 µg/ml. Western blot analysis revealed that RGZ treatment increased the expression of Bax and cleavage-caspase 3 and reduced the expression Bcl-2 and caspase 3 proteins. FCM demonstrated that RGZ could increase the apoptosis rate of the HepG2 cells, with a concentration of 40 µg/ml producing the greatest effect. GW9662 suppressed the effects of RGZ on the Bax, cleavage-caspase 3, Bcl-2 and caspase 3 protein expression in the HepG2 cells. These results are consistent with the hypothesis that RGZ is able to induce apoptosis in HepG2 cells. In concordance with these results, a number of previous studies have reported that RGZ induces apoptosis in leukemia K562 and cholangiocarcinoma QBC939 cells (19,20). Together, these results suggest that RGZ may be a novel therapeutic agent for the treatment of liver cancer in the clinical setting. However, there is a lack of evidence with regard to the effect of RGZ treatment on HCC *in vivo*. The anti-HCC effects of RGZ therefore require further investigation.

To ascertain the molecular mechanisms by which RGZ induces the apoptosis of HepG2 cells, its effect on PPAR-γ activation were examined. Unexpectedly, RGZ and GW9662 treatment did not affect PPAR-γ expression. However, administration of RGZ was observed to upregulate p-PPAR-γ expression, while GW9662 reduced the expression of p-PPAR-γ. This may be as RGZ activates PPAR-γ, rather than increasing its expression, leading to the apoptosis of the HepG2 cells.

A number of studies have demonstrated that PPAR-γ activation has a therapeutic effect on cancer cells that depends on the induction of apoptosis (20). Therefore, pathways downstream of PPAR-γ associated with the regulation of apoptosis, including PI3K/Akt signaling, were examined next in the present study. It has been demonstrated PI3K/Akt signaling is important in inducing anti-apoptotic effects in chronic myeloid leukemia (21) and that PPAR-γ activation can regulate PI3K/Akt signaling (22). In the present study, PPAR-γ activation had no effect on the expression of the PI3K regulatory subunit, p85, however, it did attenuate p85 activation, as shown by the significantly lower levels of p-p85 in the RGZ-treated HepG2 cells compared with the control cells. This result prompted the investigation of Akt activity, as p-p85 leads to Akt activation. In line with the aforementioned results, RGZ significantly decreased the Akt activity, as indicated by the downregulation of p-Akt with no effect on the expression of Akt. Taken together, these data suggest that RGZ may treat liver cancer cells by enhancing PPAR-γ activation, through which PI3K/Akt signaling activation is suppressed, thus inducing apoptosis.

The PI3K/Akt pathway has long been recognized to be important in regulating the immune response (23,24). Different PI3K heterodimers control cell survival, proliferation, B- and T-cell receptor signaling and chemotaxis in B and T lymphocytes (23,24). More recently, it has been reported that the PI3K/Akt pathway has versatile roles in apoptosis in various cell types, including K562 cells, lung cancer cells, monocytes, macrophages and parenchymal cells (25,26). Therefore, the present study conducted an additional investigation into the effect that blocking PI3K/Akt signaling had on apoptosis in the HepG2 cells. Given the capacity of RGZ treatment to induce apoptosis, it is worth noting that PPAR-γ activation induced by RGZ may be involved in additional pathways other than the PI3K/Akt signaling, such as the MAPK kinase cascade (27). As a result, further studies focusing on the pathways associated with PPAR-γ activation during the induction of HepG2 cell apoptosis are necessary.

In summary, the current study presents evidence that RGZ affects the induction of apoptosis in HepG2 cells *in vitro*, and that the mechanism involves the stimulation of PPAR-γ to suppress PI3K/Akt signaling activation. Therefore, RGZ may be a promising therapy for the treatment of liver cancer in the clinical setting.
